# Syncope without prodromes during fever in type 1‐induced BrS patient: Looks can be deceiving

**DOI:** 10.1002/joa3.12889

**Published:** 2023-06-28

**Authors:** Vincenzo Russo, Mirella Limatola, Corina Valentina De Santis Ciacci, Alfredo Mauriello

**Affiliations:** ^1^ Department of Medical Translational Sciences University of Campania “Luigi Vanvitelli”, Monaldi Hospital Naples Italy

**Keywords:** Brugada syndrome, programmed ventricular stimulation, syncope, tilting test

A 53‐year‐old man, with no significant past medical history or family history of sudden cardiac death (SCD), was admitted to the Emergency Department for transient of loss consciousness (TLOC) and facial trauma. The TLOC occurred at rest, in an upright position, without prodromes and/or specific triggers. The patient was not taking any drugs or substances that could have explained the TLOC. At admission, the body temperature was 37.8°C. The blood tests and the chest radiography suggested the diagnosis of bacterial lobar pneumonia. Transthoracic echocardiography (TTE) showed no cardiac abnormalities. The electrocardiogram (ECG) showed a coved type downward ST‐segment elevation in V1 and V2 followed by a negative T wave, compatible with the type 1 Brugada pattern (Figure 1). The patient was referred to Cardiology Unit for arrhythmic risk stratification. After 2 days of hospitalization, with the fever disappearing and the normalization of inflammatory status, a transition from type 1 to type 2 Brugada ECG pattern occurred (Figure 2). No electrocardiographic risk markers for ventricular fibrillation (VF) were present. The late potentials on the signal‐averaged electrocardiogram (SAECG) were negative. No arrhythmic events were recorded at the in‐hospital telemetric monitoring.

**FIGURE 1:**
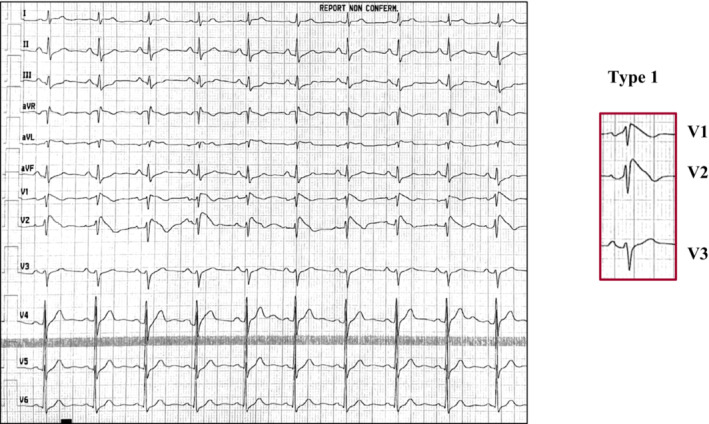
Twelve‐leads electrocardiogram at admission shows the type 1 Brugada pattern. The ECG shows a coved type downward ST‐segment elevation in lead V1 and V2 followed by a negative T wave.

**FIGURE 2:**
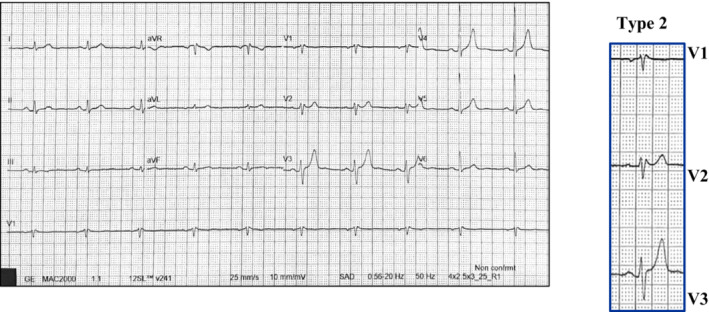
Twelve‐leads electrocardiogram at fever disappearing shows type 2 Brugada pattern. The ECG shows a distinct r′ wave ≥2 mm in lead V1–V2 with subsequently ≥0.5 mm STE and a positive or biphasic T wave in V2 that creates a “saddle back” morphology.

The clinical interpretation of TLOC without prodromes in patients with the type 1 Brugada pattern was not univocal and its nature has been debated (arrhythmic or not), such as the patients' management (conservative or invasive). Head‐up tilt test (HUTT) performed according to the Italian Protocol showed the induction of vasodepressive syncope without prodromes, characterized by a sudden fall in blood pressure with increased heart rate (Figure 3). An endocardial three‐dimensional (3D) map of the right ventricle was constructed using a high‐resolution mapping system (Rhythmia Hdx™ Mapping System, Boston Scientific Corporation, Marlborough, MA, USA) and the programmed ventricular stimulation (PVS) at two ventricular sites, right ventricular apex (RVA), and right ventricular outflow tract (RVOT), with up to three premature extrastimuli, before and after flecainide 2 mg/kg over 10 min, was performed. At unipolar (cutoff: 5.5–8.3 mV) and bipolar (cutoff: 0.2–1 mV; 0.5–1.5 mV) voltage mapping in sinus rhythm and at the propagation map, no abnormalities were shown. The PVS was negative for the induction of ventricular arrhythmias. Finally, some doubts remained about the etiology of syncope, and a long‐sensing vector loop recorder (Biomonitor IIIM, Biotronik, Berlin, Germany) was implanted. The patient was discharged with the diagnosis of vasodepressive syncope in need of physical counterpressure manoeuvres and tilt training. At 12 months follow‐up, there were no arrhythmias at loop recorder monitoring or syncopal recurrence. The next‐generation sequencing‐based gene panel test for cardiovascular genomics, including the SCN5A gene, was negative for BrS pathogenic variants.

**FIGURE 3:**
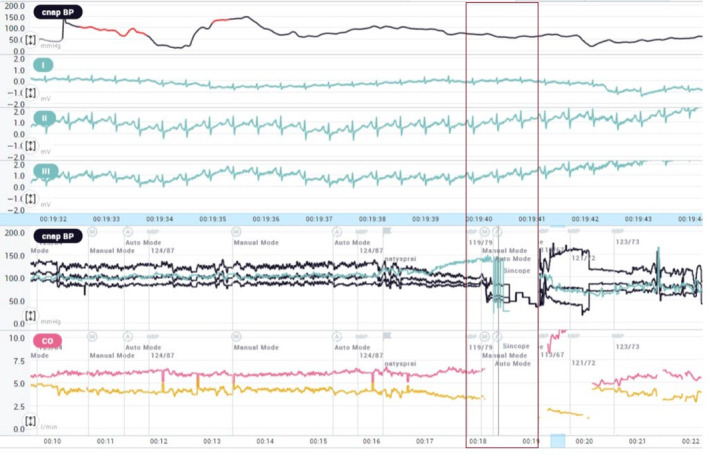
Noninvasive beat‐to‐beat monitoring of blood pressure by TaskForce Touch Cardio (CNSystems, Graz, Austria) during head‐up tilt test. The red box shows the blood pressure drop (black lines) and the increased heart rate (teal line) during syncope.

The evaluation of syncope in BrS patients, especially in those with type 1‐induced ECG pattern, represents a challenging issue since a differential diagnosis from neurally mediated and arrhythmic forms is mandatory to optimize clinical management. The clinical presentation of syncope may be not sufficient for distinguishing neurally mediated from arrhythmic syncope. Indeed, specific triggers and typical prodromes may be present in both forms of syncope among patients with channelopathies and may often precede the nonarrhythmic syncope.^1^

Although in BrS patients, there is a high incidence of neurally mediated syncope, the etiology of syncope is difficult to determine in up to 30% of BrS patients.^2^ A systematic review of nine studies including 1347 BrS patients affected by syncope and stratified according to the clinical evaluation in suspected arrhythmic syncope, undefined syncope, or neurally mediated syncope showed an annual incidence of malignant arrhythmic events of 2.8%, 2.2%, and 0.7%, respectively.^3^ In our patient, the clinical presentation of syncope suggested a suspected arrhythmic syncope since the event occurred during fever and had abrupt onset without prodromes and triggers. However, the relatively low predictive value of the clinical diagnosis of suspected arrhythmic syncope led us to perform an accurate multiparametric assessment to stratify the arrhythmic risk. In this perspective, HUTT may be useful to confirm the diagnosis of vasovagal syncope, but it cannot rule out an associated arrhythmic form. The HUTT‐induced reproducibility of spontaneous symptomatology should be assessed to consider the events caused by common underlying causes. Moreover, the PVS showed a high negative predictive value among patients with type 1‐induced Brugada pattern, but a lower positive predictive value, regardless of the history of syncope.^4^ According to our in‐hospital protocol, we use the 3D endocardial mapping of the right ventricle to evaluate the electroanatomic substrate of patients with type 1‐induced BrS pattern at baseline and during the PVS‐induced arrhythmias.^5^

The use of an implantable loop recorder (ILR) should be considered in patients with Brugada pattern and unexplained syncope^6^; and in BrS patients with low/intermediate risk, it can be helpful in guiding the management and ascertaining the cause of unexplained syncope.^7^

Implantable cardioverter‐defibrillator (ICD) implantation should be limited for patients with type 1‐induced Brugada pattern with documented arrhythmic syncope^6^ since the risk of lead infection and inappropriate ICD therapies is not negligible.^8^

Syncope without prodromes represents a challenging clinical issue in patients with channelopathies. A careful comprehensive evaluation aiming to exclude the correlation between arrhythmic events and clinical symptoms should be considered in the patients' centered care of subjects with type 1‐induced BrS pattern.

## FUNDING INFORMATION

This research received no specific grant from any funding agency in the public, commercial, or not‐for‐profit sectors.

## CONFLICT OF INTEREST STATEMENT

Authors declare no conflict of interests for this article.
